# School Dropout and Adolescent Homicides in São Paulo: A Case-Control Study on Programmatic Vulnerability

**DOI:** 10.1007/s10935-026-00916-8

**Published:** 2026-04-20

**Authors:** Marcelo Ryngelblum, Maria Fernanda Tourinho Peres

**Affiliations:** Departamento de Medicina Preventiva, Faculdade de Medicina, Universidade de São Paulo, Av. Dr. Arnaldo, 455 – Cerqueira César, São Paulo, 01246-903 SP Brazil

## Abstract

Adolescent homicide is a major public health challenge in Brazil. This study investigates the association between school dropout and homicide death in São Paulo, exploring justice-system involvement as a potential mediator. A population-based case-control study was conducted. Cases (*N* = 939) were adolescents (10–19 years) killed by homicide (2015–2020), identified via police records and linked to school databases. Controls (*N* = 939) were randomly selected after stratification of the cases by sex. Data were analyzed using conditional logistic regression to estimate Odds Ratios (OR). Mediation analysis estimated natural direct (NDE) and indirect effects (NIE) through court proceedings. Most victims were male (94.7%) and out of school at the time of death (68.8% males; 50.0% females). In adjusted models, school dropout was strongly associated with homicide for both males (ORadj = 5.54; 95% CI 4.33–7.09) and females (ORadj = 5.23; 95% CI 1.60–17.07). For males, a history of court proceedings showed a high association with homicide (ORc = 16.74; 95% CI 12.75–21.98). Mediation analysis indicated that court proceedings accounted for 24% of the total association (NIE OR = 1.48; 95% CI 1.34–1.64). The proportion mediated was higher for males (25%) than for females (14%). School dropout is a significant marker of vulnerability associated with adolescent homicide. Although justice-system involvement explains part of this association, particularly for males, the direct association remains predominant across sexes. These results indicate that school disengagement and conflict with the law are interlinked exploratory pathways in the trajectories of lethal victimization among adolescents.

Keywords: Homicide, Adolescent, Vulnerability, Secondary Data Analysis, Data Linkage, Violence

.

## Introduction

Brazil, like many countries in the Global South, faces significant challenges related to urban violence and the education of its citizens. In 2023, the country recorded approximately 46,000 homicides, with half of the victims being young people aged up to 29 years (FBSP, 2024). Data from the 2022 school census indicates that approximately 9.8 million young people (aged 15–29) dropped out of school without completing basic education, representing 19.9% of this age group (FRM, 2024). This data highlights educational gaps that directly impact the country’s social and economic development and categorize Brazil as a country with low educational attainment, where approximately 40.9% of individuals aged between 25 and 64 have not completed basic education (OECD, 2023).

Among the primary risk factors for violence, whether lethal or non-lethal, none appears to have as significant an impact as low educational attainment, which perpetuates cycles of poverty and inequality and exacerbates the social and economic marginalization of vulnerable groups (Falbto el al., 2001; Gawryszewski & Costa, 2005; Sant’Anna et al., 2005; Leite, 2008; Cole & Gramajo, 2009; Teixeira, 2011; Borges & Alencar, 2015; Cerqueira, 2016; UNICEF, 2017).

Recently, the APHA (2018) has argued that school dropout—defined as a student’s failure to continue studies and complete the basic school cycle—should be considered a public health issue, given that educational disparities are a primary indicator of health inequalities. Lower levels of educational attainment affect health and have a cascade effect on the lives of children and adolescents, impacting their employment and income opportunities, involvement in crime, incarceration, and, notably, their mortality.

In the municipality of São Paulo, Brazil’s largest and wealthiest city, data from the 2022 School Census indicates that dropout rates were 5.3% in secondary schools and 1.6% in the final years of elementary school within the public network (INEP, 2023). This data highlights the magnitude of the dropout problem and the significant educational challenges.

In the Brazilian context, school dropout is a crucial factor in the trajectories of adolescents who are victims of homicide, significantly contributing to their vulnerability. Studies show that approximately 79% of adolescent homicide victims in Porto Alegre had already dropped out of school prior to their deaths (Sant’Anna et al., 2005). Similar results were observed in the state of Ceará, where approximately 70% of homicide victims had been out of school for at least six months (UNICEF, 2017). A case-control study conducted in Recife with adolescent homicide victims revealed that only 77% of cases were enrolled in school at the time of the incident (Falbo et al., 2001). These results underscore the complex intersection between school dropout and lethal violence among young people, highlighting the need for intersectoral research to understand vulnerabilities to violent death.

Although several studies, particularly in the US and Europe, have addressed the issue of school dropout in relation to a range of negative outcomes, such as criminal involvement (Thornberry et al., 1985), drug use and mental health problems (Maynard et al., 2015), and unemployment (Ramsdal et al., 2013), we have not found any studies that specifically address the risk of homicide victimization in general, or during adolescence. Among studies from countries in the Global North, school dropout is frequently addressed as a risk factor for criminal involvement and committing violent acts (Maynard et al., 2015; Dennison, 2020). It is important to argue that in the Brazilian and Latin American contexts, which exhibit the highest homicide rates globally (UNODC, 2023), school dropout presents specific characteristics. It is often associated with youth vulnerability and exposure to urban violence, rendering young people more susceptible to homicide victimization.

Our study, in turn, posits that school dropout represents a violation of the rights of children and adolescents, and is therefore a failure of the state. The problem of school dropout and early exposure to violence will be analyzed through the lens of programmatic vulnerability (Ayres, 2009), which refers to the structural failures of state policies in achieving their objectives and guaranteeing rights. Considering school dropout from these perspectives implies recognizing the barriers and structural deficiencies that compromise educational permanence and violate the right to education, especially in contexts where students are exposed early to lethal violence and adverse socioeconomic factors.

In this article, we reiterate the centrality of school in the experience of adolescence by providing not only formal knowledge but also social and emotional skills essential for personal development, as it serves as the primary institution for sociability and building repertoires at this stage of life. If the emergence of adolescence is strictly linked to the schooling process (Santos, 1996), we question to what extent a common experience of adolescence exists in the Brazilian context, marked by extreme social inequality where social groups are excluded early from the schooling process and exposed to severe violence.

Drawing on a case-control study that utilized secondary data from various departments and employed the record linkage methodology, our objective is to investigate the association between school dropout and the risk of homicide among adolescents (10 to 19 years old) in the Municipality of São Paulo (MSP) between 2015 and 2020, specifically examining the mediating role of criminal involvement in this association.

## Theoretical Model and Hypothesis

**Fig. 1:**
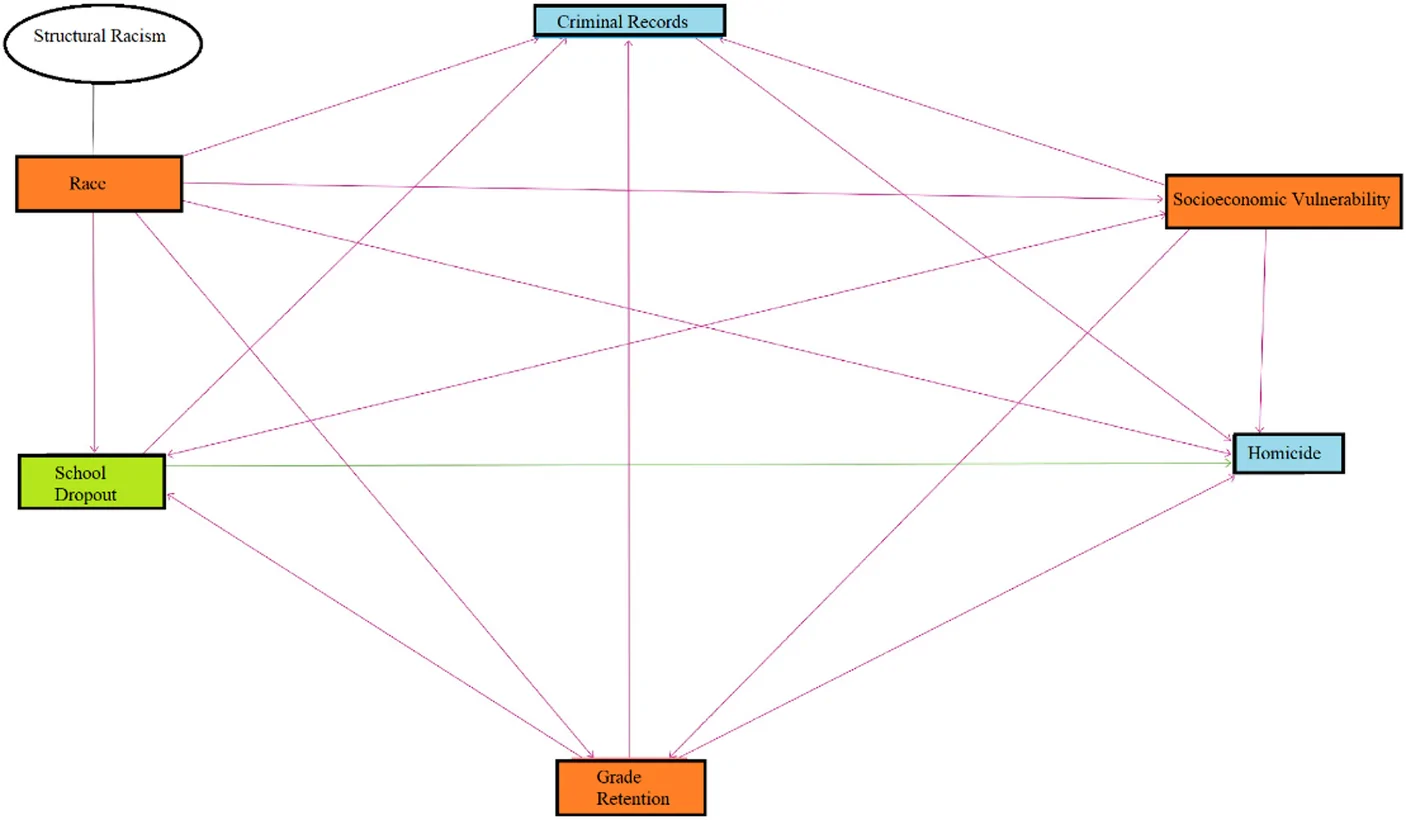
Explanatory model of the association between dropping out of school and death by homicide among adolescents

The association between school dropout and the odds of homicide was analyzed using the theoretical model presented in Fig. 1. The primary objective is to investigate the association between school dropout and death by homicide. Black, Brown, and Indigenous (as in Portuguese “Pretos, Pardos e Indigenas” - PPI) adolescents exhibit higher odds of school dropout (FRM, 2024), which may be understood as a manifestation of structural racism (an unobserved variable) or as being linked to social vulnerability. Within the context of structural racism, PPI adolescents face systemic challenges that extend beyond socioeconomic precariousness, being associated with increased likelihood of school retention (UNICEF, 2021; FRM, 2024) and having criminal records (Ribeiro & Benelli, 2017), especially among Black and Brown individuals. Furthermore, the disproportionate burden of homicide death among PPI adolescents is well-documented (FBSP, 2024).

Socioeconomic vulnerability is associated with higher odds of school dropout and grade retention (FRM, 2024), conflict with the law (Orth & Bourguignon, 2021), and death by homicide (Tavares et al., 2015). Grade retention, in turn, is linked to increased odds of school dropout (Rebelo, 2009), a history of criminal record (Machado et al., 2021), and violent death (CPPHA, 2024). According to the presented model, school dropout is associated with increased odds of having a criminal record, which is further associated with the likelihood of death by homicide; thus, a criminal record is investigated as a potential mediating variable. Consequently, our model explores both a direct association between school dropout and homicide and an exploratory indirect pathway mediated by justice-system involvement.

## Method

### Design

This study adopts a population-based case-control design. It was conducted by linking public administration databases (Health, Education, Public Security, Justice, and Social Assistance), which enabled the creation of a unified database with individualized information. The record linkage methodology was employed to link the data. After consolidating the databases, all analyses were conducted using a single anonymized dataset. The data were accessed between October 2022 and December 2024. Further information on the technical procedures for database construction can be found in Ryngelblum (2025).

### Inclusion Criteria and Sources of Information

#### Cases

A database containing all homicides in the Municipality of São Paulo (MSP), as recorded by the São Paulo State Public Security Department (SSP-SP) based on police reports (BOs), was provided by the São Paulo Committee for the Prevention of Homicide in Adolescence (CPPHA). All adolescents aged 10 to 19 years who died by homicide between 2015 and 2020 in the MSP were selected as cases, totaling 1,036 homicide records. Homicides were defined as all cases resulting in the adolescent’s death by intentional violence, classified by police officers as: Intentional homicide, Death in the context of a Robbery, Bodily Injury Followed by Death, and Death Resulting from Police Intervention. The SSP-SP database was linked to the individual database of the Mortality Information System (SIM), which allowed for the qualification of victims’ sociodemographic information. Both SIM and SSP are primary sources of information on violent mortality in Brazil.

#### Controls

Using individualized data from the São Paulo State Department of Education (SEE) (2002–2020), we conducted a population-based case-control study. Controls were randomly selected from the nearly universal pool of students first enrolled in public Elementary School I (2002–2016) in the Municipality of São Paulo, after stratification of cases by sex. To ensure chronological and historical comparability, the control group was drawn from the same birth cohorts and school entry years as the cases, ensuring they were exposed to similar educational policies and socioeconomic contexts. Exposure status (school dropout) was defined based on the last recorded school status prior to the outcome event for cases, and throughout the equivalent developmental period for the control group. All educational trajectories were longitudinally monitored until the end of 2020.

### Variables

#### Main Independent Variable (Exposure)

School dropout was considered to have occurred when: (a) the school enrollment record was classified as a dropout with no subsequent school enrollment and no record of school cycle completion; or (b) when the student did not complete the school cycle and no subsequent school enrollment or enrollment transfer was recorded. Rather than a situational event, this variable identifies dropout as the culmination of a longitudinal process of educational disengagement, serving as a marker of a cumulative trajectory of vulnerability that precedes the outcome.

#### Independent Variables for Adjustment

- **Sociodemographic**: age, sex (male and female), and race/color (Black, Brown, Indigenous, White, and Asian).
- **Socioeconomic vulnerability** was measured using two variables:

**(a) Family in poverty (yes/no)**: measured by the family’s registration in CadÚnico, a national system for registering families or individuals in poverty, managed by the Municipal Secretariat for Social Assistance and Development (SMADS).

**(b) Family in a situation of extreme poverty (yes/no)**: measured by the family’s registration in CadÚnico as a beneficiary of the Bolsa Família Program (PBF). The PBF is a federal government cash transfer program that benefits families in situations of extreme social vulnerability.

- **History of school failure (Yes/No)**: defined through the record of student failure in the SEE-ESP database.

#### Mediating Variable

History of criminal record exerts a mediating effect on the association between school dropout and death by homicide in our model. In our study, two variables express this history, and one of them will be selected for the final mediation model. These are:

1. **History of Juvenile detention (Fundação Casa) (yes/no)**: The Department of Justice and Citizenship, through Fundação Casa, provided information on the juvenile detention history of all adolescents included in the study.
2. **History of court proceedings (yes/no)**: The São Paulo Court of Justice provided information on the initiation of court proceedings for all adolescents.

### Analysis

Initially, owing to the high proportion of missing data for the race/color variable among controls, a multiple imputation model was utilized to estimate the missing values. The imputation was carried out in Stata. Multiple datasets were generated and subsequently combined to obtain robust estimates and reduce the bias introduced by the missing data. For the analysis of the race/color variable, we decided to recategorize it as binary, with the categories defined as follows: 0) White and Asian, and 1) Black, Brown, and Indigenous (PPI). The decision to include the Indigenous population along with the Black and Brown population aims to recognize and address the particular experiences of inequality and marginalization faced by these groups.

We conducted a descriptive analysis of the data, calculating measures of central tendency and dispersion for the quantitative variables, and proportions and 95% confidence intervals for the categorical variables. The crude association between each variable and the outcome was analyzed using simple logistic regression models, calculating the crude Odds Ratio (ORc) and respective 95% confidence intervals. Based on the results of this analysis, we selected the socioeconomic vulnerability and history of criminal record variables for inclusion in the final mediation model.

Logistic regression models were constructed separately for (I) exposure and mediator, (II) mediator and outcomes, and (III) exposure and outcome, for the total sample and stratified by sex. These models were adjusted for age, socioeconomic vulnerability, race/color, and grade retention.

The mediation analysis was conducted in Stata/BE 18.0. We estimated the natural direct effect (NDE), the natural indirect effect (NIE), the total effect (TE), and the proportion of the effect of school dropout on the risk of homicide that was mediated by criminal record (history of prosecution in the justice system). An interaction term between the exposure variable and the mediator was included in the total and male models. In the female group, only 7 women had a criminal record, and among those 6 dropped out from school. Sparsity and high overlap between school dropout and criminal record resulted in insufficient unique variance and power to estimate moderated mediation effects, rendering the model unidentified. A pure mediation model was assumed for the female group.

All analyses were stratified by sex, adjusted by age, race/color, grade retention, and socioeconomic vulnerability.

### Ethical Considerations

The study was approved by the Research Ethics Committee of the Faculty of Medicine of the University of São Paulo (FMUSP) (process 5.647.223) and by the São Paulo Municipal Health Department (process 5.683.265). All departments involved in the study granted authorization for data access and agreed to conduct the research. In addition, the research was approved by the Judiciary of the State of São Paulo, ensuring the necessary ethical and legal compliance.

## Results

Among the 1036 homicides recorded by the SSP-SP, 939 were identified in the SEE-ESP database after linkage (90.64%). Of the identified cases, 94.68% (*N* = 889) were male and 5.32% (*N* = 50) were female. During the period from 2015 to 2020, three deaths were recorded among the controls, and the underlying causes of these deaths did not involve interpersonal violence. The sociodemographic characteristics of the adolescents are presented in Table 1. The ages of cases and controls are very similar, with an average of 17 years. There is a notable difference in the racial composition between male cases and controls, with a predominance of PPI among homicide victims compared to controls (67.38% vs. 42.97%). This difference is much smaller among females (46% vs. 34%). In both male and female cases, there were higher percentages across all vulnerability markers. For male victims, a history of court proceedings (60.74%) and a history of juvenile detention (42.86%) are particularly prominent.

**Table 1:** Characteristics of cases and controls by sex, São Paulo (2015–2020)

	Total (*n* = 1878)		Male (*n* = 1778; 94.68%)		Female (*n* = 100; 5.32%)	
	Case (*n* = 939)	Control (*n* = 939)	Case (*n* = 889)	Control (*n* = 889)	Case (*n* = 50)	Control (*n* = 50)
Age						
Mean(SD); Median (IQR)	17.41 (1.41); 18 (17–19)	17.89 (1.72); 18 (17–19)	17.44(1.3); 18 (17–19)	18 (1.73); 18 (17–19)	17 (1.62); 17 (16–18)	17.23 (1.25); 17 (17–18)
	N (%)		N (%)		N (%)	
*Race*						
White	313 (33.33)	418 (44.52)	286 (32.17)	391 (43.98)	27 (54.00)	27 (54.00)
Asian	0	12 (01.28)	0	11 (01.24)	0	1 (02.00)
Black	87 (09.27)	30 (03.19)	80 (09.00)	30 (03.37)	7 (14.00)	0
Brown (mixed-race)	535 (56.98)	294 (31.31)	520 (58.49)	278 (31.27)	15 (30.00)	16 (30.00)
Indigenous	1 (00.11)	6 (00.64)	0	5 (00.56)	1 (02.00)	1 (02.00)
No information	3 (00.32)	179 (19.06)	3 (00.34)	174 (19.57)	0	5 (10.00)
*Binary race (after imputation)*						
White/Asian	313 (33.33)	540 (57.50)	286 (32.17)	507 (57.03)	27 (54.00)	33 (66.00)
Black/Brown/ Indigenous (PPI)	626 (66.67)	399 (42.50)	603 (67.38)	382 (42.97)	24 (46.00)	18 (34.00)
*Independent variables*						
Poverty	540 (57.51)	386(41.11)	509 (57.26)	360 (40.49)	31 (62.00)	26 (52.00)
Extreme poverty	268 (28.54)	80 (08.52)	257 (28.91)	75 (08.44)	11 (22.00)	5 (10.00)
Court proceedings	546 (58.15)	76 (08.09)	540 (60.74)	75 (08.44)	6 (12.00)	1 (02.00)
Juvenile detention	383 (40.79)	25 (02.66)	381 (42.86)	25 (02.81)	2 (04.00)	0
Grade retention	609 (64.86)	379 (40.36)	585 (65.80)	370 (41.62)	24 (48.00)	9 (18.00)
School dropout	637 (67.84)	220 (23.43)	612 (68.84)	214 (24.07)	25 (50.00)	6 (12.00)

Various signs of school disengagement are noticeable before dropout, especially grade retention. In Table 1, the disparities between the case and control groups in terms of grade retention are evident, with both male (65.80%) and female (48.00%) cases showing higher percentages compared to the controls. In the male group, most retention was attributed to school attendance issues, with percentages varying between cases (61.82%) and controls (50.39%). Among female adolescents, retentions among the cases were mainly due to school performance issues (60%). Among female controls, 50% of retentions were for performance and 50% for insufficient attendance (data not shown in the table). Table 1 also shows the distribution of school dropout among cases and controls, by sex. A significant proportion of adolescent homicide victims were truant at the time of death, among males (68.84%) and females (50%), demonstrating very high percentages compared to controls.

The association between study variables and homicide is presented in Table 2. The crude analysis indicates a positive association between school dropout and homicide victimization among adolescent males (ORc = 6.98; 95% CI 5.66–8.61). For females, the point estimate suggested a similar direction (ORc = 7.33; 95% CI 2.65–20.28), although this result should be interpreted as an exploratory and less precise estimate due to the lower sample size and wider confidence interval.

A history of grade retention also increases the odds of homicide in the crude analysis among males (ORc = 2.69; 95%CI [2.22, 3.26]) and females (ORc = 4.21; 95%CI [1.69, 10.44]). Among males, the PPI population exhibited higher odds of homicide death compared to White and Asian individuals. (ORc = 2.80; 95%CI [2.30, 3.40]). Although the odds of homicide appeared higher for PPI adolescents in the female group, this finding was not statistically significant (ORc = 1.65; 95%CI [0.73, 3.71]). Additionally, socioeconomic vulnerabilities are also associated with an increased odds of homicide among males and females. Among males, living in poverty (ORc = 1.97; 95%CI [1.63, 2.38]) and extreme poverty (ORc = 4.48; 95%CI [3.39, 5.91]) were associated with higher odds of death. Among females, although both ORc values suggest a greater odds of death, the results were not statistically significant. The magnitude of the association was notably high for a history of prosecution in the justice system (ORc = 16.74; 95%CI [12.75, 21.98]) and juvenile detention (ORc = 25.82; 95%CI [16.98, 39.27]) among males (Table 2).

**Table 2:** Crude association between study variables and death by homicide among adolescents from municipality of São Paulo (2015–2020)

	Crude odds ratio (ORc)	*p*-value	95% CI
*Total*			
History of dropping out of school	6.91	< 0.001	5.63; 8.47
Age	0.81	< 0.001	0.76; 0.86
Race/color (Ref: White/Asian)	2.71	< 0.001	2.24; 3.26
Families in poverty	1.94	< 0.001	1.62; 2.33
Families in extreme poverty	4.34	< 0.001	3. 31; 5.70
Court proceedings	15.73	< 0.001	12.03; 20.56
Juvenile detention	25.09	< 0.001	16.51; 38.12
School retention history	2.72	< 0.001	2.25; 3.28
*Male*			
History of dropping out of school	6.98	< 0.001	5.66; 8.61
Age	0.80	< 0.001	0.76; 0.86
Race/Color (Ref: White/Asian)	2.80	< 0.001	2.30; 3.40
Families in poverty	1.97	< 0.001	1.63; 2.38
Families in extreme poverty	4.48	< 0.001	3.39; 5.91
Court proceedings	16.74	< 0.001	12.75; 21.98
Juvenile detention	25.82	< 0.001	16.98; 39.27
School Retention History	2.69	< 0.001	2.22; 3.26
*Female*			
History of dropping out of school	7.33	< 0.001	2.65; 20.28
Age	0.87	0.315	0.67; 1.14
Race/Color (Ref: White/Asian)	1.65	0.222	0.73; 3.71
Families in poverty	1.51	0.313	0.68; 3.34
Families in extreme poverty	2.54	0.109	0.81; 7.94
Court proceedings	6.68	0.084	0.77; 57.69
Juvenile detention	–	–	–
School Retention History	4.21	0.002	1.69; 10.44

**Fig. 2:**
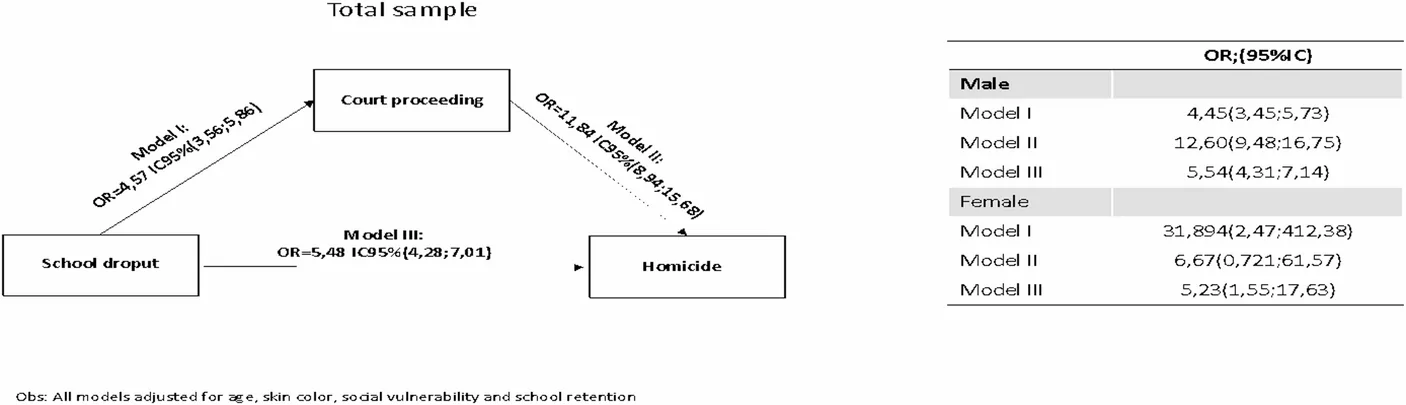
Association between school dropout and court proceedings, court proceedings and homicide, and school dropout and homicide, municipality of São Paulo (2015–2020). (Note: All models adjusted for age, socioeconomic vulnerability, and grade retention.)

Figure 2 presents the results of the regressions for school dropout and court proceedings (Model I), court proceedings and homicide (Model II), and school dropout and homicide (Model III) for the total sample and stratified by sex. All models are adjusted for age, race/color, social vulnerability and school retention. School dropout was positively associated with the odds of court proceedings among both males and females. The magnitude and low precision of the ORadj among females is striking (ORadj = 31.89; 95% CI [2.47, 412.38]) and is probably explained by the small number of cases and controls. The results, however, are statistically significant and indicate increased odds of court proceedings among females who dropped out of school. We also found a higher odds of homicide among adolescents with a history of court proceedings, whether they were males (ORadj = 12.60) or females (ORadj = 6.67). The result for females was not statistically significant. A positive and significant association was also found between school dropout and homicide for both sexes, with ORadj = 5.54 for males and ORadj = 5.23 for females.

**Fig. 3:**
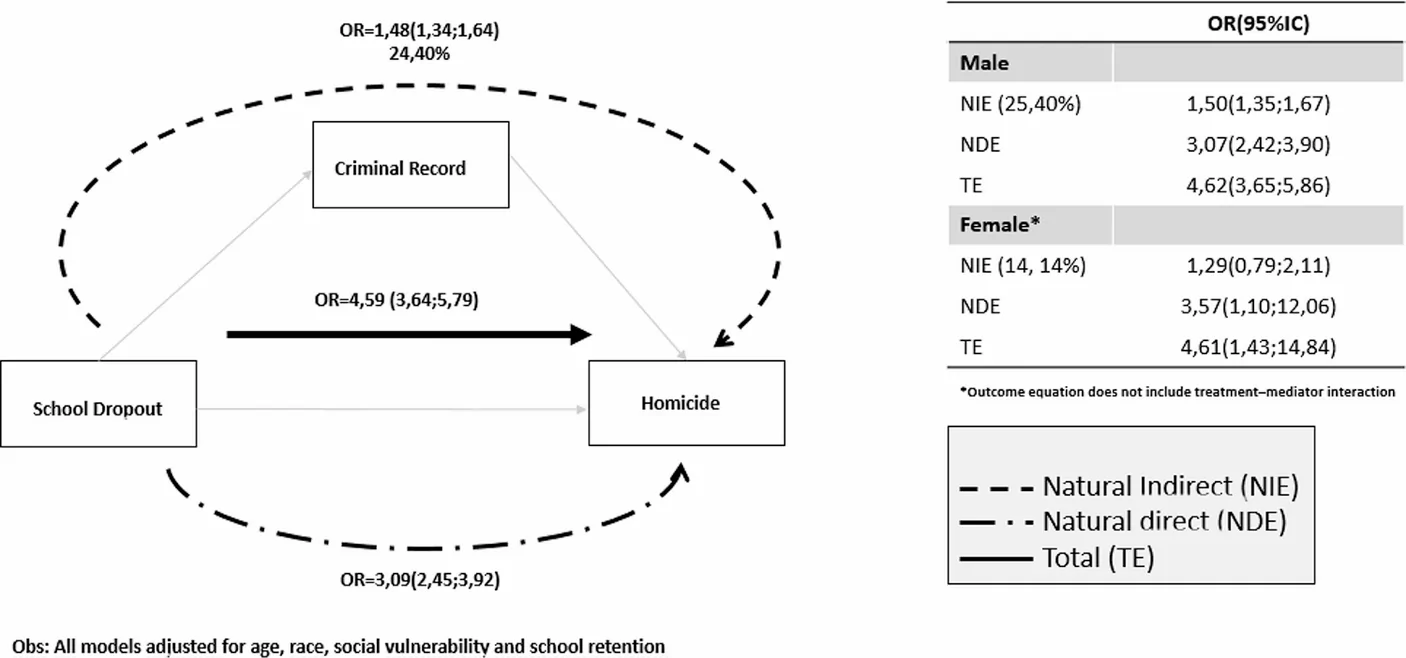
Mediation analysis between school dropout and conflict with the law, conflict with the law and homicide, and school dropout and homicide, municipality of São Paulo (2015–2020). (Note: All models adjusted for age, socioeconomic vulnerability, and grade retention.)

The results of the adjusted mediation analysis are presented in Fig. 3. The total effect of school dropout exhibited a positive association with homicide victimization. In the mediation model, school dropout was associated with 4.6 times higher odds of being a victim of homicide. The natural direct effect (NDE) was statistically significant (ORadj = 3.09), suggesting that school dropout is independently linked to the outcome. Additionally, the natural indirect effect (NIE) was also positive (ORadj = 1.48; 95% CI 1.34–1.64), indicating that court proceedings accounted for 24% of the total association between school dropout and homicide.

For males, the proportion mediated by the NIE was 25%. While the direction of the estimates for females appeared similar, the low statistical precision and sample size for this group require a cautious interpretation. The NIE accounted for 14% of the association among females; however, this path appeared less prominent than for males, suggesting that for this subgroup, the link between school dropout and homicide may involve factors outside the justice system. These sex-specific pathways should be considered exploratory, particularly for females, where the lower number of cases may limit the identification of precise mediation effects.

## Discussion

The analysis of homicides and their potential factors revealed patterns in the association between school dropout and adolescent victimization in São Paulo. The data show that school dropout is positively associated with the odds of homicide among male and female adolescents, an association that persists even after adjusting for covariates. Part of this association is accounted for by a history of court proceedings (25% among males, 14% among females). The direct path represents the majority of the estimated association, reinforcing the importance of school dropout as a factor linked to adolescents’ vulnerability to intentional violent death and highlighting the necessity of further investigation into other potential mediators.

The results show similar patterns in the association between school dropout and homicides, demarcated by sex, where school dropout appears to be a relevant factor in the death trajectories, especially for female adolescents. The sex difference is notable concerning the proportion NIE accounts for in male (25%) and female (14%) adolescents. These results suggest that school dropout may play a more prominent role for female adolescents through pathways less directly related to the justice system, although these sex-specific findings should be interpreted as exploratory due to the limited sample size for females.

The analysis revealed that the vast majority (67.84%) of homicide victims were out of school at the time of death, a finding consistent with similar studies conducted in other Brazilian cities (Falbo et al., 2001; Sant’Anna et al., 2005; UNICEF, 2017). School dropout is a complex and multifactorial phenomenon, linked to the intersection of social, economic, and institutional factors that contribute to the social marginalization of adolescents. As Dubet (2003) points out, schools, which should be attractive to adolescents, promote inclusion and provide access to cultural and educational resources, can act as agents of social exclusion, thereby exacerbating inequalities. The school institution can perpetuate exclusion by reproducing social inequalities, such as the scarcity of resources and opportunities, selectivity in access, among other factors. These factors not only marginalize the most vulnerable students but also contribute to a continuous cycle of exclusion and inequality.

Furthermore, the impact of structural racism exacerbates disparities in school enrollment and retention among Black and Indigenous adolescents. These systemic inequities heighten exposure to programmatic vulnerability, specifically increasing the risk of violent victimization (FBSP, 2024). In this context, programmatic vulnerability must be analyzed through a racial lens; structural racism dictates the distribution of resources and institutional precariousness, disproportionately affecting racialized groups. Ultimately, school dropout and exposure to lethal violence in Brazil are not merely coincidental but are inextricably linked to a racialized social order that perpetuates educational exclusion and insecurity.

To better understand the phenomenon, it is argued that school dropout should be discussed not only as an exclusive failure of the school institution or as a reflection of personal and family deficiencies, but as one of the facets of the failure to protect children and adolescents in Brazil. School dropout can only occur when various child protection mechanisms also fail, indicating that the problem is not only related to school but also extends beyond its walls. Economic difficulties, health and justice problems, or other social and psychological issues are directly linked to the ability to continue and engage in school. Thus, to address school dropout, it is necessary to critique and reformulate institutional mechanisms that fail to guarantee rights, offer equitable opportunities, and reduce programmatic vulnerabilities (Ayres et al., 2009).

It is worth noting that, far from being a homogeneous phenomenon, school dropout encompasses numerous nuances that deserve consideration. The primary factors associated with school dropout in Brazil are the need to generate income and a lack of interest in the school process (Neri, 2015). In a field study conducted in São Paulo, Rodrigues (2023), based on interviews and ethnography, details the pathways to school dropout, indicating that beyond work, income generation, and a lack of interest in the school process, the experience of psychological suffering, bullying, isolation, distrust of school, engagement in new types of entrepreneurship, as well as the feeling of not learning anything from school and the loss of a reference person in the school process, all correlate with school trajectories. In her view, the issue should be conceptualized through the dynamics of approaching and distancing oneself from the school environment, which is marked by ambiguities, doubts, and moral conflicts, rather than an indistinct, binary idea of abandonment or dropout. This perspective allows for a procedural and complex understanding of the phenomenon. It should be emphasized here that the progressive processes of disengagement with the school environment can indicate signs of disinterest that ultimately culminate in school dropout (Jarjoura, 1996; Bridgeland et al., 2006; Rodrigues, 2023). These signs include changes in student behavior, with occasional delays, academic losses, and absences, progressing to intermittent participation in school activities, extended periods of absence, or even an entire school cycle, ultimately culminating in school dropout itself. If identified in a timely manner, these signs can help prevent school dropout.

One aspect that stands out in our study and merits future investigation is the association between the justice system, via court proceedings and passage through juvenile justice institutions (Fundação Casa), and the dynamics of violence in adolescence, which is linked to significantly higher odds of homicide. The findings highlight the relevance of conflict with the law as a partial mediating mechanism in the relationship between school dropout and homicides, offering important implications for interventions aimed at reducing violence. Involvement in criminal activities is often imposed on vulnerable children and adolescents as either a seemingly advantageous option or, in many cases, the only available alternative within the labor Market (Feltran, 2007; Peres, 2014; Galdeano & Almeida, 2018). Furthermore, their interaction with the justice system and the ensuing stigmatization process ultimately interfere with their developmental trajectory. The process of ‘criminal subjection’ (Misse, 2010) —where individuals, rather than their actions, are criminalized—further exposes adolescents to police violence in Brazil from an early age. This exposure was the leading cause of mortality among this population in the MSP during the analyzed period, with Black adolescents being disproportionately affected (Ryngelblum & Peres, 2025).

## Limitations

Our study has several limitations and strengths that should be considered. Of the 1,036 homicides recorded during the period, approximately 10% of the victims lacked information regarding their school enrollment and could not be linked to the homicide database, being excluded from our study. Factors contributing to the partial identification of cases in the school database include the possibility that adolescents were enrolled in educational institutions in other states, significant errors in school enrollment records which compromised data linkage, and the absence of schooling records for some victims. Sensitivity analysis, however, showed no significant differences between linked and unlinked cases for sex and race. Age differences between groups were significant (*p* = 0.0004). It should be noted that 90% of all recorded cases were analyzed, which reduces the possibility of selection bias. It is also necessary to highlight the low quality of racial information in SEE-SP’s records, with approximately 20% of students having no information. Missing values were estimated using multiple imputation techniques.

Our model explores both a direct association between school dropout and homicide and an exploratory indirect pathway mediated by justice-system involvement. In relation to the timeline, school dropout antecedes both criminal record and homicide. We accounted for sequential ignorability by including key demographic and socioeconomic controls. Unfortunately, considering our data are administrative we can not assure all potential confounders were included in our analysis and that adjustments were sufficient to block all backdoors. To evaluate the impact of unmeasured confounders in our results we run a sensitivity analysis using a Bivariate Probit model. A high correlation between error terms was observed (rho ˜=1, *p* < 0.001) indicating that a hidden bias is present and sequential ignorability was not fully met. The robustness of the association between the mediator and the outcome (beta = 1.22, z = 23.06, *p* < 0.001), however, suggests that even in the presence of unmeasured confounders, the mediation path can be considered a valid hypothesis. Additionally we run a probit model decomposition using the Karlson-Holm-Breen(KHB)-Method. Both direct and indirect paths are highly significant (z = 9.84, *p* < 0.001/ z = 14.47, *p* < 0.001) indicating a robust mediational logic. Total effect, direct effect and indirect effect were respectively beta = 0.91486, beta = 0.565, and beta = 0.34977. Aproximately 38% of the effect of school dropout in homicide is explained by adolescent conflict with the law. So, even considering the presence of hidden bias, these results suggest the logic and assumption of our mediation analysis are robust.

The results, however, should be interpreted with caution. Although the Natural Direct and Indirect Effects offer valuable insights into potential pathways, they rely on the assumption of no unmeasured confounding between exposure, mediator, and outcome—an assumption that was not met in our study. Consequently, these results are framed as exploratory associations and statistical pathways within a synergistic trajectory of vulnerability, rather than definitive causal mechanisms.

Furthermore, the lack of granular dating for the onset of justice-system involvement relative to the exact moment of school disengagement prevents the absolute exclusion of reverse causality. We propose, however, that these factors represent a synergistic process of exclusion rather than a strictly linear sequence. Additionally, while territorial variables were available, their inclusion was beyond the scope of this specific analysis, which may result in residual confounding from unmeasured neighborhood-level factors. Finally, although race/color was utilized as a proxy for social disadvantage, we acknowledge that individual-level data cannot fully capture the structural complexity of racism. Therefore, these factors are addressed as the broader contextual landscape that shapes trajectories of lethal vulnerability.

This is the first population-based case-control study on adolescent homicides using linkage to official databases conducted in Brazil, one of the most violent countries in the Americas and globally. Our literature review did not identify another similar study on adolescent homicides. Although studies using secondary data are subject to issues with information quality, we consider this approach to be a significant strength of this study, not only because it minimizes memory bias but, more importantly, because it allowed us to include all cases that occurred in the MSP and to select a control group from a database with universal coverage in the MSP.

## Final Considerations

The analysis of adolescent homicides in São Paulo reveals that school dropout serves as a notable marker of vulnerability associated with victimization. Our findings indicate that school dropout is positively associated with the odds of homicide among both sexes, with point estimates suggesting potential pathways for both males and females. Although justice-system involvement—via court proceedings—accounts for a portion of this association, particularly among males, the direct association remains prominent across the sample. These results suggest that school disengagement and conflict with the law function as interlinked exploratory pathways within the trajectories of lethal victimization. Rather than establishing definitive causal mechanisms, this study highlights the complex institutional failures and structural inequalities that characterize the school-to-violence pipeline in the Brazilian context, pointing to the relevance of further research into intersectoral protective mechanisms.
